# Acoustic analyses of the RAVDESS corpus of emotional stimuli

**DOI:** 10.1121/10.0042364

**Published:** 2026-02-04

**Authors:** Devon P. Major, Monita Chatterjee

**Affiliations:** 1 Center for Hearing Research, Boys Town National Research Hospital, Omaha, Nebraska 68131, USA; 2 Department of Communication Sciences and Disorders, Northwestern University, Evanston, Illinois 60208, USA

## Abstract

Freely available online emotional stimuli allow researchers to conduct emotional perception research without needing to record their own and easily compare findings across studies; however, the acoustic properties, specifically the prosodic cues, are frequently unreported. Prosodic cues are important for a listener to contrast between the talker's emotional tone. Thus, understanding how these cues differ among an emotional stimuli database allows for a nuanced interpretation of findings for emotion perception researchers. This paper analyzes the prosodic cues (fundamental frequency, duration, and loudness) of the speech stimuli in *The Ryerson Audio-Visual Database of Emotional Speech and Song (RAVDESS).*

## 1. Introduction

Emotional communication is an important part of daily conversation: misinterpreting emotions in social communication can have a negative impact on one's psychosocial well-being and quality of life.^1^ A number of different clinical populations exhibit prosody-related deficits in accurately interpreting spoken emotions during communication, including individuals with hearing loss who listen with a cochlear implant^2,3^ and autistic individuals.^4^ Despite the importance of affective verbal communication in human interactions, research on the facial and acoustic cues used by humans to express and comprehend emotions has been relatively limited.

Using multimodal emotional stimuli that are readily available in online databases allows researchers to conduct emotional perception research without having to record their own emotional stimuli and readily compare findings across studies using the same stimulus set. To search for emotional stimulus set to use in an experiment, researchers can use the KAPODI,^5^ an online site that allows for an easy search of 364 online available databases that are freely available or available upon request.^6^ However, the acoustic properties of the stimuli available in individual databases are frequently not reported. This poses a barrier for researchers who are seeking to understand the specific acoustic features of emotions that the population that they are studying might be having difficulty dealing with or for those studying how acoustic features of emotions are impacted by different acoustic environments.

In the auditory domain, a talker's emotion is conveyed through lexical-semantic and prosodic cues, but prosodic cues have been shown to dominate.^7,8^ In many available online databases, creators choose to use sentences that are semantically neutral in emotion, leaving primarily prosodic cues available to the listener for emotion recognition tasks. Prosodic cues include changes in voice pitch (*f*_o_), timbre, loudness, and duration/speaking rate: these covary in specific ways to signal individual emotions. For example, *anger* and *happiness* are characterized by an increase in mean *f*_o_, *f*_o_ variability, and intensity, as well as a quicker rate of speech from neutral speech. *Fear* is also associated with an increase in speech rate (shorter sentence duration) and increase in mean *f*_o_, whereas *sadness* is associated with a decrease in mean *f*_o_, *f*_o_ range, and intensity, as well as longer duration.^9–12^ Emotion-specific prosodic cues are also likely to differ based on the talker and directions given for eliciting the emotional speech token. Therefore, when using emotional speech stimuli in research designs, it is important to know their specific acoustic characteristics as well as how these acoustic characteristics differ between the stimuli.

The goal of the current paper is to illustrate how prosodic cues change as a function of stimulus-related factors (talker sex, repetition, emotion, sentence, and emotional intensity level) across emotional stimuli by analyzing the speech stimuli in one of the available online databases that is commonly used in auditory research, *The Ryerson Audio-Visual Database of Emotional Speech and Song (RAVDESS)*, provide a description of the prosodic cues (standard deviation of *f*_o_, mean *f*_o_, duration and loudness) as well as how they change as a function of emotion.

### 1.1 RAVDESS

The RAVDESS is a dynamic, multimodal set of facial and vocal expressions in North American English. The stimuli are recorded in an audiovisual condition from 24 professional actors [*M* = 26.0 years; standard deviation (SD) = 3.75; age range = 21–33 years old; 12 males and 12 females]. Actors self-identified as Caucasian (*N* = 20), East-Asian (*N* = 2), and mixed (*N* = 2, East-Asian Caucasian, and Black-Canadian First Nations Caucasian). All actors were working in Toronto, ON, Canada, and recorded the stimuli with a *neutral* North American accent. The stimulus set provides audio-only, auditory-visual, and visual-only versions of the stimuli. The emotional stimuli were recorded in speech and song.^13^ Only the speech stimuli are analyzed in this paper.

Two semantically *neutral* sentences were used (“Kids are talking by the door.” and “Dogs are sitting by the door.”). The creators of the stimulus set selected these two statements because they are both seven syllables in length and matched in word frequency and familiarity. The sentences were video recorded in eight different emotional tones*: neutral*, *calm*, *happy*, *sad*, *angry*, *fearful*, *surprise*, and *disgust*. The *calm* and *neutral* sentences are considered to be baseline conditions. All emotions, except *neutral*, were portrayed with two levels of emotional intensity: *normal* and *strong*. Actors repeated each vocalization twice. Stimuli included 60 distinct spoken utterances from each actor.^13^ Therefore, a total of 1440 stimuli of the initial stimulus set are analyzed here [talker (12) × sex (2) × statement (2) × emotion (7) × emotional intensity level (2) × repetition (2)] + *neutral* [talker (12) × sex (2) × statement (2) × repetition (2)].

This database of emotional stimuli was validated by 247 individuals with an additional 72 participants providing test-retest reliability. All stimuli have high levels of emotional validity, and test-retest intra-rater reliability scores were reported. All stimuli are freely available under a Creative Commons license and can be downloaded online. For more information regarding initial stimuli design, recording, post stimuli processing, and validation, see Refs. 14 and 15.

### 1.2 Purpose

The objective of this paper is to provide a detailed acoustic analysis of prosodic cues in the seven emotions portrayed in the RAVDESS stimulus set and how these cues differ as a function of emotion, talker sex, sentence, repetition, and emotional intensity level. We analyze and compare the variation of *f*_o_, the average *f*_o_, average intensity, variation of intensity, and duration between the seven emotions (*calm*, *happy*, *sad*, *angry*, *fearful*, *surprised*, and *disgusted*), normalized to the talker's *neutral* emotion, by talker, talker sex, emotional intensity level (strong vs normal), statement (dogs vs kids), and repetition. Specifically, the present study addresses the following research question: *to what extent do f*_o_ *variation, mean f*_o_*, mean intensity, intensity variation, and duration differ across seven emotions, and how are these prosodic properties influenced by talker sex, emotional intensity level, sentence, and repetition in video recorded speech stimuli?* We expect a main effect of emotion for all prosodic cues, suggesting that prosodic cues will differ between emotions. Specifically, based on previously conducted acoustic analyses,^9–12^ we predict that emotions produced monotonously, such as *sad*, will be longer in duration and have less variation in intensity and *f*_o_ compared to highly emotive sentences, such *as happy*, *angry*, *surprised*, and *fear*. We expect that there will be a main effect of emotional intensity level, with the *strong* sentences containing greater acoustic variation in loudness and *f*_o_, greater mean *f*_o_, and longer durations. We also anticipate a significant interaction between emotion and emotional intensity level; whereas the prosodic cues will be exaggerated (greater) for the *strong* intensity level compared to the *normal* intensity level for all emotions, we do not anticipate there to be any change across levels for the *calm* emotion. We do not expect a main effect of sentence, talker sex, and repetition. We do not anticipate an effect of talker sex because the acoustic cues are normalized to the individual talker's *neutral* sentence, thus, accounting for pitch and intensity differences between males and females. Additionally, we expect that incorporating a random effect of talker will account for a significant amount of variation in the prosodic cues as talkers naturally differ in how they use the acoustic cues to express emotions.

## 2. Methods

### 2.1 Acoustic analyses

Acoustic analyses were performed in Praat.^16^ Values for intensity and pitch contours were extracted every 0.01 s. To derive the pitch contour, a Praat script proposed by Daniel Hirst was used; this script uses an automatic estimation of minimum and maximum *f*_o_.^17^ This estimation improves the accuracy of pitch calculations by dynamically adapting the *f*_o_ range to the specific sound. Once the *f*_o_ range is determined, the pitch contour is calculated using Praat's “to pitch” method. Intensity and pitch contour extraction from the 1440 emotional stimuli were conducted using batch processing. The duration of stimuli was extracted using a custom script in matlab,^18^ which automatically detected speech onset and offset for each video. Speech onset occurred at ∼1 s and speech offset occurred before or at 4 s for all videos. If the automatic selection of speech onset or offset was determined to be incorrect via visual inspection due to noise in the stimulus waveform, manual selection of stimulus onset/offset was conducted. Extracted pitch and intensity tiers as well as duration were first analyzed in matlab and the following parameters were computed for each sentence: mean *f*_o_ in semitones normalized to the talker's mean *f*_o_ of their *neutral* sentence, standard deviation of *f*_o_ in Hz (*f*_o_ SD) normalized to the talker's mean *f*_o_ of their *neutral* sentence, sentence duration in seconds normalized to talker's *neutral* sentence, standard deviation of loudness (SD loudness, defined as the SD of root-mean-square (RMS)-based intensity values in dB), and mean loudness (quantified as RMS energy; Leq, time-averaged level in dB) relative to talker's *neutral* sentence. For each emotional sentence, all values were normalized by the talker's *neutral* sentence spoken in *normal* intensity. In the present study, the term “loudness” is used to describe RMS-based acoustic intensity derived from Praat intensity contours rather than perceptual loudness measures. Additionally, mean *f*_o_ was expressed in semitones and normalized to the mean *f*_o_ of each talker's neutral sentence to facilitate perceptually meaningful comparisons across talkers. To obtain a measure of dispersion of the *f*_o_ *values* around the mean, we also computed the standard deviation of the *f*_o_ across each utterance and normalized the mean *f*_o_ of each talker's neutral sentence. The standard deviation was not computed on a logarithmic or semitone scale because there is not a clear perceptual correlate of the variance of *f*_o_. Further, such a transformation would distort the natural distribution of *f*_o_ values around the mean. Rather, our interest was in the actual variation of the values around the mean. Normalizing to the neutral sentence's mean *f*_o_ ensured scaling to the talker's natural mean pitch.

### 2.2 Statistical analysis of acoustic cues

Statistical analysis and graphs were created in *R* version 4.4.2.^19^ Linear mixed-effects models were created using the package *lme4*.^20^ Separate linear mixed effect models were constructed for each acoustic cue: *f*_o_ SD, mean *f*_o_, duration, and SD loudness. Models were conducted on the acoustic measures to investigate the effects of emotion, talker sex, statement (kids vs dogs sentence), emotional intensity level, and repetition with a random intercept of talker and slope of emotion [acoustic measure (dependent variable) ∼ (emotion + sex + sentence + emotional intensity level + repetition)^3^ + (1 + emotion | talker)]. The random slope of emotion by talker was considered in the full model because it was anticipated that the acoustic cues would differ by talker for each emotion. Candidate models were created by simplifying the full model and splitting the data by emotional intensity level. The best-fitting model was selected using the Bayesian information criterion (BIC). To compare models that included emotional intensity level as a fixed effect (pooled models) with models that did not (split models, which divided the dataset into *normal* and *strong* subsets), the BIC values were handled differently. For pooled models, a single BIC value was computed, but for split models, a separate BIC value was obtained for each split model, and then the BICs were summed to yield a total BIC. This allowed for comparison across pooled and split strategies.

All predictors were coded using sum contrasts. Emotion (seven levels) was coded such that each coefficient reflected the deviation of an emotion from the grand mean across all emotions. Sex was coded as −0.5 and 0.5 for males and females, respectively; therefore, the main effect of sex tests for the mean difference between groups centered around zero.^21^

The *performance*^22^ package was used to check model diagnostics, including visualizing histograms. *Post hoc* pair-wise comparisons of the estimated marginal means were conducted using the *emmeans* package,^23^ with Tukey's honest significant difference (HSD) method applied to correct for multiple comparisons. *The ggplot2* package^24^ was used to create all visualizations of data.

## 3. Results

Descriptive statistics for each unique combination of independent variables' (e.g., talker 12, female, *happy*, sentence 1, repetition 1, and normal emotional intensity level) acoustic measures and best-fit model summary statistics are found in the supplementary material with the analysis of variance (ANOVA) results of the best-fit model. For *f*_o_ standard deviation and mean *f*_o_ (3.1 below) and mean loudness (3.2 below) model fit indicated that two separate models (one for each emotional intensity level) with random slope of talker (for *f*_o_ SD) and random slope of talker by emotion (average *f*_o_ and loudness) fit the data best. These models did not include intensity level as an independent variable. Additional figures depicting significant main effects and interactions are included in the supplementary material with a color-coded matrix of the estimated marginal mean differences between emotional pairs for each acoustic variable.

### 3.1 f_o_

For *f*_o_ standard deviation, fixed effects explained 36% of the variance (marginal *R*^2^ = 0.36), and the full model explained 45% (conditional *R*^2^ = 0.45). Results revealed that for the *normal* emotional intensity level, there was a main effect of emotion [*F*(6,600) = 68.07, *p* < 0.000] and sentence [*F*(1,600) = 7.97, *p* = 0.00]. The pair-wise comparison of the differences between estimated marginal means of the emotional pairs reveals more variation of *f*_o_ across sentences recorded in a *surprised* tone compared to all other emotions. Similarly, sentences produced in an *angry*, *disgusted*, or *happy* tone contained a more variable voice pitch compared to those produced in a *calm*, *sad*, or *fearful* tone.

There were main effects of emotion [*F*(6,600) = 32.78, *p* < 0.001] and sex [*F*(1,22) = 7.22, *p* = 0.01], as well as a significant interaction between the talker's sex and emotion [*F*(6,600) = 3.30, *p* = 0.00] for the *strong* emotional intensity level. Fixed effects explained 27% of the variance (marginal *R*^2^ = 0.27), whereas the full model explained 37% (conditional *R*^2^ = 0.37) for the *strong* intensity level. The male talkers included in this database used greater *f*_o_ variation when producing the majority of the emotional sentences (Fig. S3); however, the difference in *f*_o_ variation across emotions differed by sex. Pair-wise comparisons revealed that male talkers used significantly greater *f*_o_ variation when producing an *angry* sentence compared to when producing a *fearful* sentence, whereas there were no significant differences in the variation of *f*_o_ between these two emotions for females. Conversely, females used significantly more variability in *f*_o_ when producing emotional sentences in a *surprised* tone compared to *calm*, *sad*, *disgust*, and *fearful*. The difference between the degree of *f*_o_ variability in the production of a *surprised* emotional sentence and *calm*, *disgusted*, and *fearful* was smaller but still significant for male talkers.

**Fig. 1.**
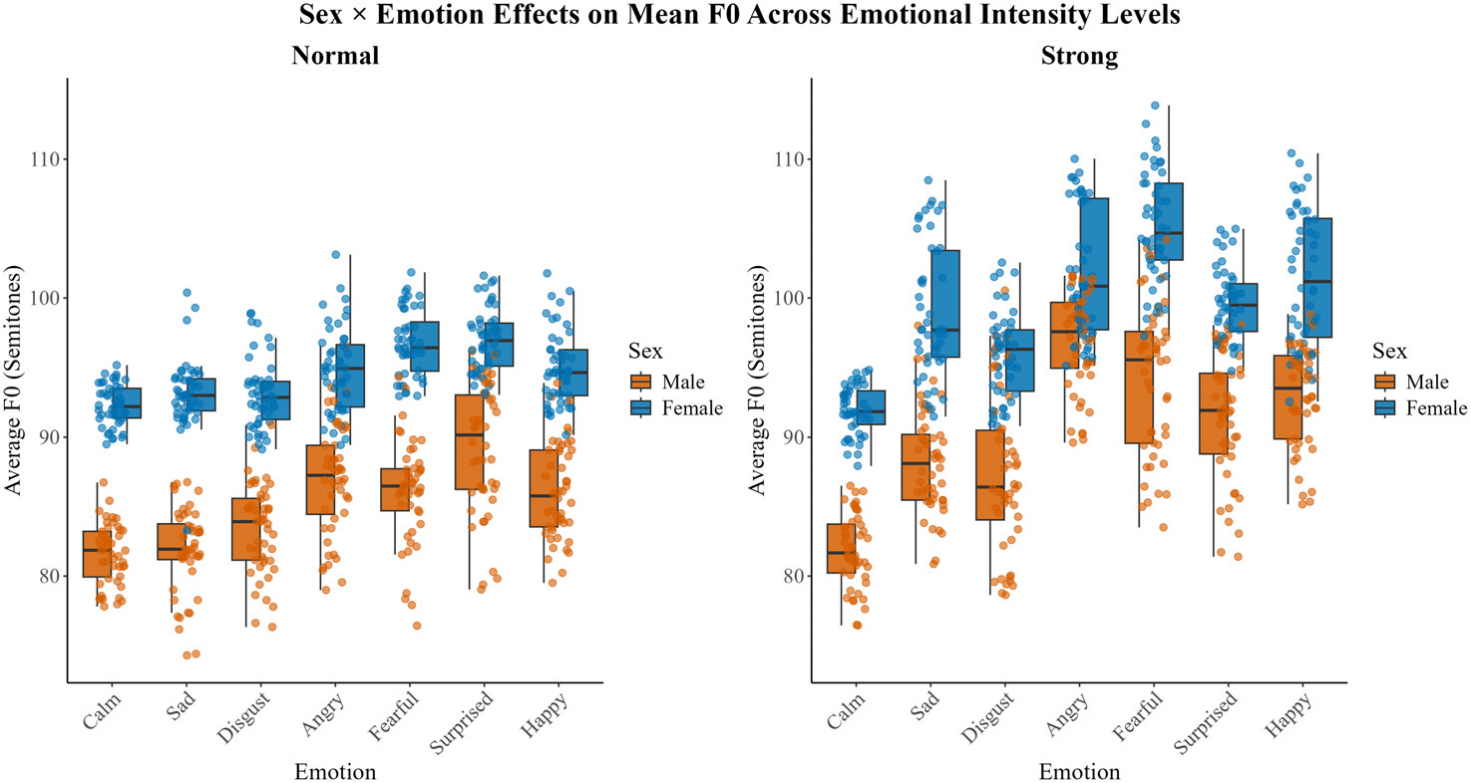
The talker sex × emotion interaction for the average *f*_o_ (in semitones) for normal and strong emotional intensity levels are depicted in (A) and (B), respectively. Each box represents the interquartile range (IQR; 25th–75th percentiles), where horizontal lines mark the median and whiskers extending to 1.5 × IQR. Individual talker values are indicated by filled-in circles. Male data are shown in orange, and female data are shown in blue. Emotions are on the *x* axis and average *f*_o_ values are on the *y* axis.

For the mean *f*_o_, *normal* intensity level, there was a main effect of emotion [*F*(6,22) = 54.21, *p* < . 001], talker sex [*F*(1,22) = 131.73, *p* < 0.001], sentence [*F*(1,468) = 5.10, *p* = 0.02], repetition [*F*(1,468) = 18.21, *p* < 0.001] as well as a significant interaction between emotion and talker sex [*F*(6,22) = 2.63, *p* = 0.04] and emotion and repetition [*F*(6,468) = 2.54, *p =* 0.02]. For the *normal* emotional intensity level, fixed effects explained 75% of the variance (marginal *R*^2^ = 0.75), whereas the full model explained 93% (conditional *R*^2^ = 0.93). The second repetition of the sentence “dogs are sitting by the door” had a significantly greater mean *f*_o_ across all emotions and talkers compared to the first repetition. Figure 1(A) depicts the difference in talker sex on average *f*_o_ across emotions for the *strong* emotional intensity level. Pair-wise comparisons indicate that for male talkers, mean *f*_o_ is significantly different between most emotional pairs, except for *happy* compared to *fearful* and *angry*, *angry* compared to *fearful*, and *sad* compared to *disgust* and *calm*. For females, the emotional pairs that are not significantly different from one another were *happy* and *angry*, *surprised* and *fearful*, *disgusted* compared to *sad* and *calm*, as well as *calm* and *sad*.

For the *strong* emotional intensity level mean *f*_o_, results revealed a significant main effect of emotion [*F*(6, 22) = 76.37, *p* < 0.001], talker sex [*F*(1,22) = 77.91, *p* < 0.001], and a significant interaction between emotion and talker sex [*F*(6,22) = 4.27, *p* = 0.005]. Fixed effects accounted for 69% of the variance (marginal *R*^2^ = 0.69), whereas the full model explained 93% (conditional *R*^2^ = 0.93). For the pair-wise comparison between emotions, there is a significant difference in mean *f*_o_ for both sexes. As evident in Fig. 1(B), female talkers have a higher normalized mean *f*_o_ compared to males across all emotions.

### 3.2 Duration

**Fig. 2.**
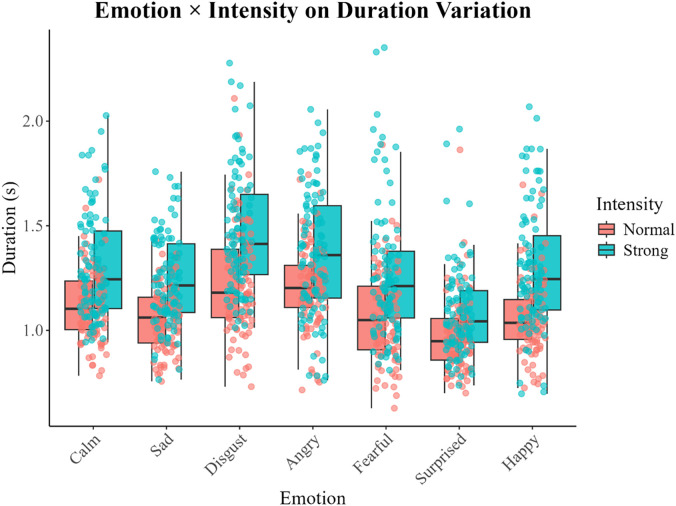
Following the same plotting configuration as in Fig. 1, the interaction between emotional intensity and emotion on sentence duration is displayed. The normal intensity level is shown in pink, and the strong intensity level appears in teal.

The full model with random slope of talker fit the duration data the best. The model indicates that duration significantly differs by emotion [*F*(6,1241) = 54.98, *p* < 0.001] and emotional intensity level [*F*(1,1241) = 308.96, *p* < 0.001], and there is a significant interaction between emotion and emotional intensity level [*F*(6,1241) = 2.56, *p* = 0.018]. Fixed effects accounted for 26% of the variance in duration variability (marginal *R*^2^ = 0.26), whereas the full model, including random intercepts, explained 52% of the variance (conditional *R*^2^ = 0.52). Visual inspection of Fig. 2 shows that duration is longer for the *strong* emotional intensity level compared to the *normal* intensity level; however, the difference in duration between the two levels differs by emotion. For example, there is less of a difference in duration between the two emotional intensity levels for sentences spoken in a *surprised* tone compared to that for a *happy* tone. There was also a significant three-way interaction between sex, sentence, and repetition [*F*(1,1241) = 5.07, *p* = 0.025] such that duration was similar between sexes for the “kid” sentence but different for the “dog” sentence in the first repetition, but in the second repetition, the pattern changed, with male talkers' duration remaining similar between the two sentences, whereas the female talkers' durations changed.

### 3.3 Loudness (RMS)

There were significant main effects of emotion [*F*(6,22) = 51.73, *p* < 0.001] and repetition [*F*(1, 468) = 16.65, *p* < 0.001] for the *normal* emotional intensity level for average loudness. For the *normal* intensity level, the model's fixed effects alone explained 51% of the variance (marginal *R*^2^ = 0.51); however, when including the talker by emotion random effect, the model explained 85% of the total variance in mean loudness (conditional *R*^2^ = 0.85), suggesting that there is a substantial number of between-talker differences.

For the *strong* emotional intensity level, there was a main effect of emotion [*F*(6,22) = 4.46, *p* = 0.004] as well as a significant interaction between sex and sentence [*F*(1,468) = 4.16, *p* = 0.042] on average loudness. For the *strong* emotional intensity level, the model's fixed effects alone explained 71% of the variance (marginal *R*^2^ = 0.71). When including the talker by emotion random effect, the model explained 92% of the total variance in mean loudness (conditional *R*^2^ = 0.92).

**Fig. 3.**
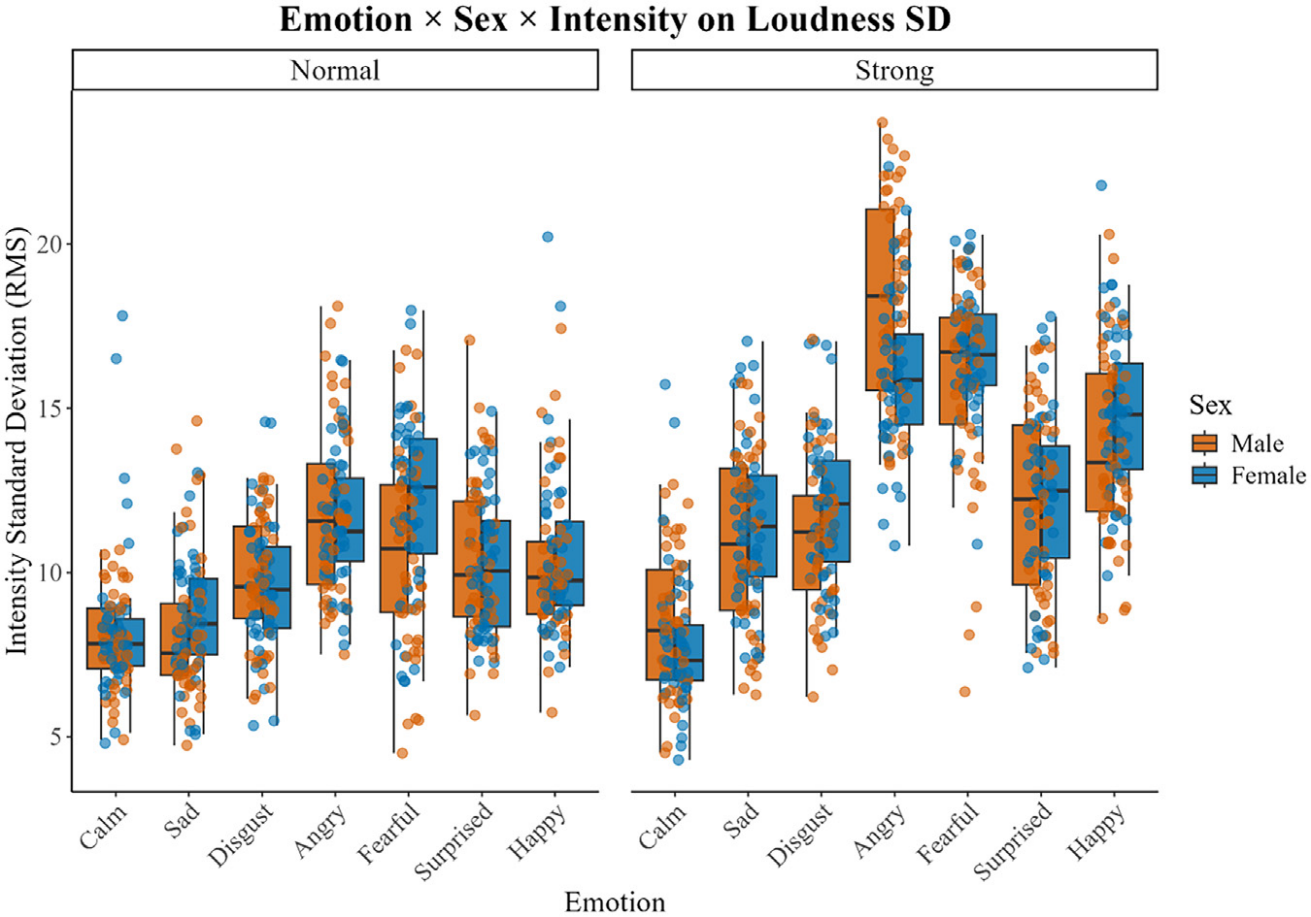
The standard deviation of intensity across the emotional sentences, separated by sex and emotional intensity levels (*normal* and *strong*) are presented to illustrate the significant three-way interaction. The plotting configuration is the same as that in Figs. 1 and 2. Male data are depicted in orange, and female data are shown in blue. The left side panel depicts the *normal* emotional intensity level data, and the right-side data depicts the *strong* emotional intensity level data.

The best-fit model for the variation of loudness was the full model with emotion by talker as a random intercept. Model results reveal a main effect of emotion [*F*(6,22) = 77.04, *p* < 0.001], emotional intensity level [*F*(1,1109) = 965.45, *p* < 0.001], and significant two-way interactions between emotion and talker sex [*F*(6,1109) = 3.55, *p* = 0.013] and emotion and emotional intensity level [*F*(6,1109) = 57.67, *p* < 0.001], which was modified in a three-way interaction between emotion, talker sex, and emotional intensity level [*F*(6,1108) = 4.11, *p* < 0.001]. Fixed effects explained 56% of the variability (marginal *R*^2^ = 0.56) and the full model, including the random intercept emotion by talker, accounted for 77% of the variance (conditional *R*^2^ = 0.77). Figure 3 illustrates the three-way interaction. For most emotions, regardless of sex, the *strong* emotional intensity levels were produced at a higher volume compared to the *normal* level, except for the “calm” emotion. For the *normal* level, differences in loudness across emotion are similar between sexes, but for the *strong* emotional intensity level, males produce sentences in an *angry* tone louder than females. Pair-wise comparisons between emotions revealed that the magnitude of the difference in loudness between emotions is greater for the *strong* emotional intensity level compared to that for the *normal* level.

## 4. Discussion

The purpose of the current study was to examine how prosodic speech cues differ by emotion, talker sex, sentence, repetition, and emotional intensity level. Importantly, this analysis characterized how multiple stimulus-related factors shape emotional prosody within a widely used emotional speech database that was video recorded. Because the same analysis used here can be extended to other affective speech databases, whereas the specific patterns may differ across databases, the present findings illustrate how emotion, emotional intensity level, semantics, repetition, and talker can influence acoustic cues in acted emotional speech stimuli and represent potential important sources of variability that are crucial for researchers to consider when selecting emotional stimuli in experimental designs and interpreting results that rely on prosodic cues. Specifically, our analysis shows several important effects and interactions.

There was a significant main effect of emotion across all acoustic variables, reinforcing the current consensus that the prosodic speech cues (variation and mean *f*_o_, intensity, and duration) play a critical role in the identification of emotion in speech. Emotions that are similar, such as *sad* and *disgusted*, had less significant differences in prosodic cues, whereas contrasting emotions, such as *happy* and *sad*, had a greater number of significant differences in prosodic cues. However, even for emotional pairs that are similar, there was at least one significant difference in prosodic cue that would aid a listener in identifying the emotion. For example, *disgusted* was significantly longer in duration than *sad*. However, d*isgusted* is a uniquely expressed emotion, and research has shown that unlike other emotions, the prosodic cues used in a talker's production of it are not correlated with a listener's ability to identify the emotion.^9^ Therefore, it is likely that a listener would rely on additional speech cues in differentiating between *disgusted* and *sad*.

Emotions typically produced with greater expressive intonation, such as *happy* and *surprised*, showed greater variation in *f*_o_ and intensity, whereas more monotonous emotions, like *sad*, exhibited less variation in these cues. In contrast to our hypothesis, *fearful* showed less variation in *f*_o_. We also predicted that more monotonous emotions would exhibit longer durations, and this was partially supported: there was evidence that *sad* had longer durations compared to surprise for the *strong* emotional intensity level but was not significantly longer than *happy* for either intensity level. Therefore, within this stimulus set recorded from actors, duration of speech was not as contrastive between monotonous and more varying pitch contours as anticipated. This could be a result of the timing instructions provided to the actors during the recordings.^13^

Emotional intensity level also influenced the acoustic speech cues. It was included as an independent variable in the duration model and variation of loudness models. In both models, there was a significant main effect of emotional intensity level, with longer durations and greater variation for the *strong* intensity, suggesting that when actors produced more expressive sentences, they emphasized these prosodic cues. Although emotional intensity levels were not statistically compared for the other acoustic cues, it is evident from comparing the trends on the graphs that the *strong* intensity level sentences were produced with greater *f*_o_ variation, higher mean *f*_o_, and greater loudness compared to those produced with *normal* emotional intensity.

There was a significant effect of sentence and repetition in some models. Specifically, the sentence type (kid vs dog) influenced the degree of *f*_o_ and loudness variation. The “kid” sentence elicited greater pitch fluctuations and greater variation in loudness than the “dog” sentence, suggesting that certain lexical-semantic content may elicit more expressive prosody, even when the sentence structure is tightly controlled. This highlights the importance of considering lexical-semantic content when designing emotional speech stimuli and interpreting acoustic outcomes. Repetition influenced the mean *f*_o_; specifically, the second repetition of the sentences had a higher mean *f*_o_ compared to the first repetition. The observed variability in mean *f*_o_ across recordings of the same emotion and sentence indicates that even trained actors may vary their fundamental frequency use within an emotion. This suggests that emotions, although contrastable by mean *f*_o_, are not expressed with absolute consistency within an emotion by the same talker. It may take practice for the actors to produce the emotion fully, thus, randomly selecting the first vs the second sentence produced could have ramifications on the acoustic properties of the stimuli used. From a theoretical standpoint, this within-emotion, within-talker variability may reflect a more nuanced reality of emotional expression.

Despite normalization of each talker's acoustic features to their *neutral* baseline, several significant interactions involving talker sex were observed. These effects suggest that normalization may not eliminate sex-related acoustic differences, particularly in intensity and *f*_o_use, which may reflect persistent anatomical or stylistic choices in vocal production. Specifically, female talkers had a higher normalized mean *f*_o_ compared to male talkers; however, male talkers produced emotional sentences with greater *f*_o_ variation. Male talkers also used greater variation in loudness compared to female talkers for the *strong* emotional intensity when producing *angry* sentences, which may reflect the stylistic choice among the male actors in the current stimulus set.

Finally, the inclusion of talker as a random effect accounted for a substantial portion of the variance across several models, confirming that individual talker differences significantly influence prosodic cue use. This finding reinforces the idea that emotional prosody is shaped not only by emotion itself but also by talker-specific style. Such variability highlights the complexity of vocal emotion expression and the need to consider emotion-specific and talker-specific sources of acoustic variation, especially when designing stimuli. These stylistic differences were observed among a stimulus set recorded from mostly self-identified white females and males. Therefore, it is likely that increasing the diversity of actors included in the stimulus set would show greater stylistic differences in prosodic cue use, which may be more consistent with the affective speech a listener hears in their daily life. Researchers should consider using diverse stimulus sets for this reason.

Taken together, the findings demonstrate that although prosodic cues reliably differentiate emotions, affective speech is shaped by emotional intensity level, sentence semantic-lexical content, repetition, and talker sex. It is important to note that these data are derived from recordings of actors portraying emotions in a video recording, using controlled semantic content and in the absence of social interactions. Thus, the results observed here reflect characteristics of acted, video recorded, emotional speech, which may contain exaggerated emotional cues relative to spontaneous emotional communication and differ from auditory-only recordings of emotional speech. When actors know they are being video recorded rather than audio recorded alone, their emotional portrayals are conveyed through vocal (auditory) and facial (visual) cues. As a result, they may differentially emphasize one modality over the other. For example, an actor may use greater vocal pitch fluctuations in auditory-only recordings compared to those used in audiovisual recordings, where they may distribute emotional expression across auditory and visual cues. Still, it remains unclear how these portrayals differ from emotional communication in real-world conversational settings. Therefore, these findings should be interpreted as characterizing prosodic variability within video recorded emotional speech stimuli from actors rather than as direct representations of emotional expression in real-world conversational settings. Nonetheless, this complexity has important implications for experimental design and theories of emotional speech. Although the specific acoustic patterns reported here are tied to the RAVDESS stimulus set, the influence of emotion, emotional intensity level, sentence, repetition, and talker sex highlight broader sources of variability that are likely to shape emotional speech stimuli in other databases as well. Researchers should account for these intricacies and provide acoustic analyses of the affective stimuli used in their studies as observed effects may be driven by acoustic properties specific to the selected stimuli.

## Supplementary Material

See the supplementary material for figures of significant main effects and significant interactions that were not included in the body of main paper, as well as figures that depict color-coded matrices of emotional pair-wise comparisons for each of the acoustic variables (SuppPub1.docx). The summary statistics for each model are found in SuppPub2.xlsx.

## Data Availability

The data that support the findings of this study are openly available in OSF at https://osf.io/qyt7d/overview?view_only=afbc2cd2ee3a4234a05548eeae5dba44, Ref. 24.
